# Anomalous Polarons in Two‐Dimensional Organometallic Perovskite Ferroelectric

**DOI:** 10.1002/advs.202406885

**Published:** 2024-09-23

**Authors:** Junhong Yu, Yadong Han, Yunfan Yang, Hang Zhang, Yi Liu, Jinlong Xu, Zhihua Sun, Jianbo Hu

**Affiliations:** ^1^ Laboratory for Shock Wave and Detonation Physics Institute of Fluid Physics China Academy of Engineering Physics Mianyang 621900 China; ^2^ State Key Laboratory for Environment‐Friendly Energy Materials Southwest University of Science and Technology Mianyang 621010 China; ^3^ State Key Laboratory of Structural Chemistry Fujian Institute of Research on the Structure of Matter Chinese Academy of Sciences Fuzhou Fujian 350002 China; ^4^ Department of Physics College of Physics and Information Engineering Fuzhou University Fuzhou Fujian 350108 China

**Keywords:** macroscopic polarizations, perovskite ferroelectric, photophysics, polarons, transient absorptions

## Abstract

The concept of ferroelectric polarons is proposed to partially explain the exceptional optoelectronic properties observed in lead halide perovskites (LHPs). It is intriguing but unclear how this proposal, which involves local or transient polarizations, applies in general to 2D LHPs with long‐range ferroelectricity. Here, this work presents a pioneering time‐domain experimental investigation of polarons in ferroelectric (IA)_2_(MA)_2_Pb_3_Br_10_ (IMPB; IA is isoamylammonium and MA is methylammonium) using transient absorption spectroscopy. Compared to non‐ferroelectric LHPs, IMPB exhibits several distinct polaronic properties closely associated with macroscopic polarizations of ferroelectricity, including a prolonged polaron formation time (≈1.1 ps), a Stark splitting of the bleaching (≈63 meV), and a giant polaron Mott density (≈7.6 × 10^18^ cm^−3^). These findings broaden the realm of 2D polaron systems and reveal the decisive role of static/unidirectional polarizations on polaron physics in 2D LHPs.

## Graphical abstract

Time‐domain experimental investigations of polarons in 2D organometallic perovskite ferroelectric (IA)_2_(MA)_2_Pb_3_Br_10_ (IA: isoamylammonium and MA: methylammonium) reveals several distinct properties closely associated with macroscopic polarizations of ferroelectricity, including a prolonged polaron formation time, a Stark splitting of the bleaching, and a giant polaron Mott density.

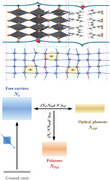

## 1 Introduction

Optoelectronic research on solution‐processed lead halide perovskites (LHPs) has experienced a significant surge in interest over the past decade, marked by a rapid escalation of record performances.^[^ ^1^, ^2^, ^3^, ^4^, ^5^, ^6^, ^7^, ^8^, ^9^, ^10^ ^]^ Among the myriad fascinating properties of LHPs, one of the most intriguing and perplexing is their ability to maintain remarkably long carrier lifetimes and diffusion lengths, despite modest mobilities and extensive disorder.^[^ ^11^, ^12^, ^13^ ^]^ This phenomenon challenges the prevailing consensus observed in conventional crystallized semiconductors like Si or III‐V materials. In attempts to elucidate these exceptional carrier properties, Zhu et al. proposed the ferroelectric polaron mechanism,^[^ ^14^, ^15^, ^16^ ^]^ suggesting that the electric field generated by charge carriers could induce local phase transitions, leading to the formation of ferroelectric nanodomains. These nanodomains could effectively mitigate Coulombic interactions, acting as a protective “shield” to reduce carrier scattering among themselves or from ionized defects and phonons. To date, the ferroelectric polaron effect has garnered substantial support from dynamic simulations^[^ ^16^, ^17^ ^]^ and static/time‐resolved spectroscopy experiments,^[^ ^17^, ^18^, ^19^, ^20^ ^]^ thereby reshaping the understanding of excited state species and exerting a significant influence on optoelectronic applications of LHPs.

Recently, the convergence of research efforts on LHPs and graphene‐like atomically thin materials has reignited considerable interest in 2D layered organic‐inorganic hybrid perovskite (OIHP) with the formula A_2_B*_n_*_‐1_Pb*_n_* X_3_ *_n_*_+1_, where B represents a small organic monovalent cation, and *n* denotes the number of lead‐halide (X) unit cells between the organic spacer cations (A).^[^ ^20^, ^21^ ^]^ In addition to the enhanced quantum confinement and reduced dielectric screening,^[^ ^22^, ^23^ ^]^ another noteworthy characteristic of 2D OIHPs is their high degree of freedom to accommodate dynamic motions of organic cations, facilitating symmetry breaking and ferroelectricity.^[^ ^24^, ^25^ ^]^ It is important to note that the ferroelectric phenomenon in 2D OIHPs differs from the above‐mentioned ferroelectric polaron proposal in two key aspects:^[^ ^15^, ^16^, ^24^ ^]^ i) it is static and permanent, not vanishing when the charge is removed; and ii) it involves macroscopic polarization with unidirectional alignment, rather than local microscopic ordering with radial symmetry centered on the charge. Consequently, 2D ferroelectric OIHPs offer an ideal platform for investigating the interplay between polaronic effects and ferroelectricity in a collective and macroscopic manner.

In this work, we present, for the first time, a direct time domain view of polaron dynamics in (IA)_2_(MA)_2_Pb_3_Br_10_ (IMPB; IA is isoamylammonium and MA is methylammonium), which exhibits bulk ferroelectricity at room temperature. Utilizing ultrafast broadband pump‐probe spectroscopy, we have observed that polaronic states in IMPB form on a longer time scale of over one picosecond (e.g., the polaron formation time is ≈0.3 ps in CH_3_NH_3_PbBr_3_^[^ ^16^ ^]^), leading to the emergence of the photoinduced absorption (PIA) band. Concurrently, the photoinduced bleaching (PIB) feature of IMPB undergoes a splitting into two distinct branches during the polaron formation process, with a detuning energy reaching ≈63 meV. Further analysis of the excitation density‐dependent polaron dynamics confirms that the Mott polaron density (i.e., the density when polaron wavefunctions start to overlap) in IMPB is one order of magnitude larger than that in non‐ferroelectric LHPs.^[^ ^25^, ^26^ ^]^ These results reveal the impact of macroscopic ferroelectricity on polaron dynamics, which produces the internal electric field and screens the many‐body Coulomb interactions, resulting in a suppressed carrier‐phonon scattering, the Stark splitting of electronic transitions, and a reduced polaron radius.

## 2 Results

**Figure 1:**
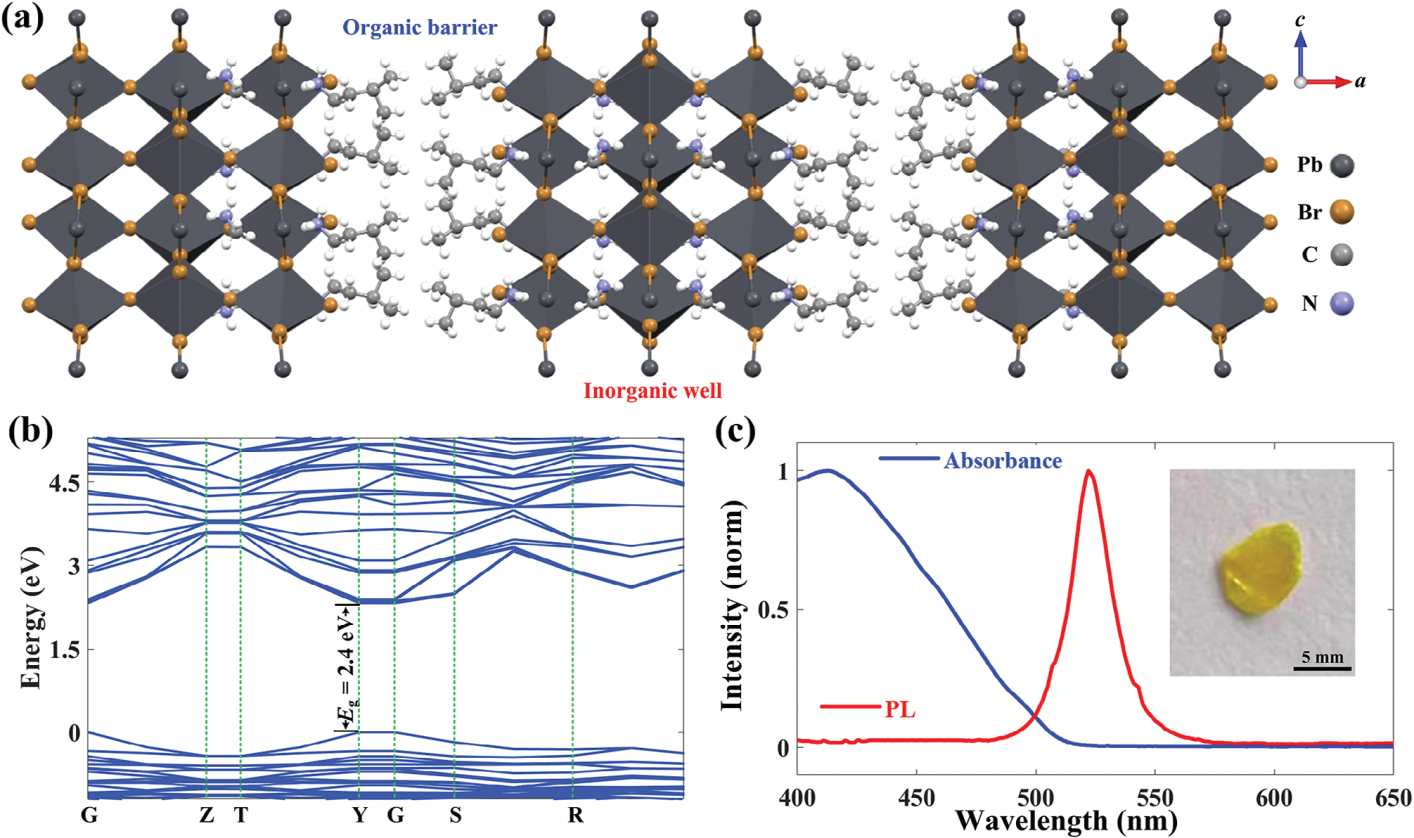
Crystal structure and optical properties of IMPB. a) Schematic representation of the quantum‐well structure projected along the *b*‐axis. Barrier: organic cation bilayers, Well: inorganic perovskite frameworks. b) Electronic band structure of IMPB modeled by the DFT calculations. c) Absorption (the blue curve) and PL (the red curve) spectra of IMPB. Inset: the photograph of bulk crystal IMPB grown from an aqueous solution.

The bulk crystal of IMPB, whose structure is sketched in **Figure** **1**a, is prepared by the temperature‐cooling method applied to its aqueous solution.^[^ ^26^, ^27^ ^]^ IMPB features the prototypical 2D‐oriented Organic‐inorganic hybrid perovskite architecture: halometallate [Pb_3_Br_10_]_∞_ trilayers, consisted of corner‐sharing PbBr_6_ octahedra and organic spacing layers of IA^+^ cations, are orientated alternatively along the crystallographic <100> direction, with the NH_3_ groups embedding inside the inorganic wells via N─H─Br hydrogen bonds. It is noteworthy that such a crystal structure with large degrees of freedom that allows dynamic motions of organic cations (i.e., cations possess a low barrier for molecular reorientation)^[^ ^26^ ^]^ and the organic‐inorganic quantum wells that deeply confine photocarriers (i.e., the width of the quantum well is about 18 Å),^[^ ^27^ ^]^ renders IMPB with ferroelectricity at room temperature. Our recent reports have demonstrated that IMPB exhibits bulk ferroelectricity below the Curie temperatures (≈303 K) with a large spontaneous polarization (*P*_s_, 5.0 µC cm^−2^).^[^ ^26^, ^28^, ^29^ ^]^ In the ferroelectric phase (see the schematics in Figure S1, Supporting Information), the positive and negative charge centers are shifted by the reorientation of organic moieties and the distortion of PbBr_6_ octahedra respectively, which synergically give rise to the electric polarization along the *c*‐axis. We have calculated the band structure of IMPB along the <001> direction performed within density functional theory (DFT, see details in Experimental Section) and as shown in Figure 1b, the calculated direct bandgap energy (*E*_g_) is about 2.4 eV. The steady‐state photoluminescence (PL) spectrum of IMPB shows a distinct peak at ≈522 nm (see Figure 1c) and *E*_g_ of ≈2.42 eV can be estimated based on a Tauc plot of the absorption spectrum in Figure S2, Supporting Information, which agrees well with our simulated results. Please note that the ≈44 meV PL Stokes shift is considerably larger than that of II‐VI group semiconductors (e.g., CdSe colloidal nanocrystals), which can be attributed to the inherent polaronic character in lead halide perovskite ionic lattice.^[^ ^22^, ^30^, ^31^, ^32^ ^]^ Importantly, unlike the asymmetric line shape toward lower energies in different forms of 2D LHPs (single crystals,^[^ ^33^ ^]^ polycrystalline films,^[^ ^34^ ^]^ and nanocrystals^[^ ^22^ ^]^), IMPB exhibits a nearly symmetric PL spectrum, implying the absence of defects and vacancies induced interstitial bands.

**Figure 2:**
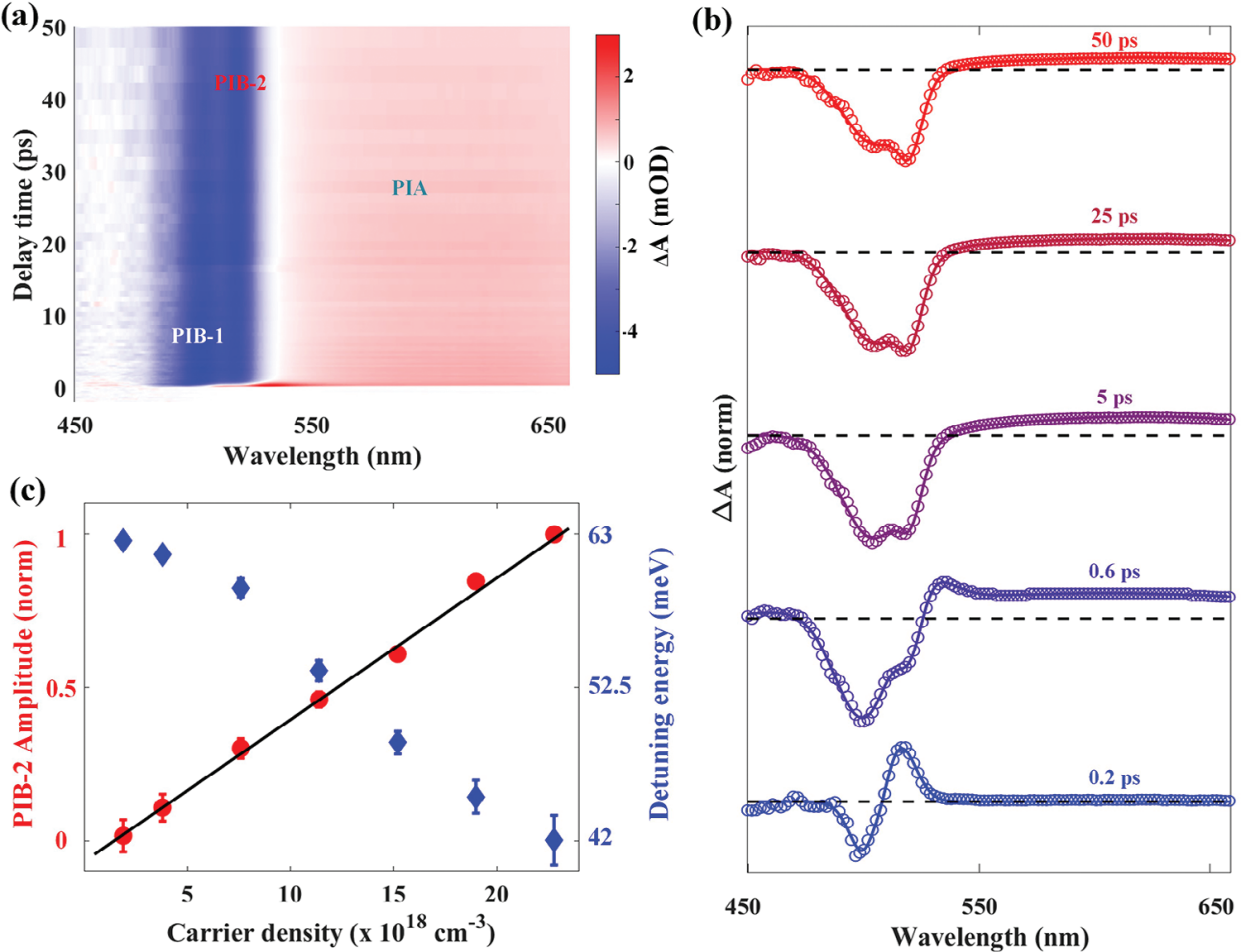
Transient absorption study of IMPB with the excitation carrier density of ≈1.9 × 10^18^ cm^−3^ and the excitation wavelength of 360 nm. a) A 2D color plot of the transient absorption spectrum with a linear vertical axis. b) Representative spectra cut at different time delays (the circles). The solids line is the Viogt fitting to extract the detuning energy between PIB‐I and PIB‐2 features. c) Left axis: Maximum TA signal of PIB‐2 component (red dots) as a function of photoexcited carrier density and its linear fitting. Right axis: the carrier density‐dependent detuning energy, which is defined as the energy difference between the splitting peaks (blue diamonds).

With the above bandgap photoexcitation (360 nm) of moderate carrier density (*N*_0_: 1.9 × 10^18^ cm^−3^), carrier dynamics in IMPB are investigated using ultrafast transient absorption (TA) spectroscopy. **Figure** **2**a shows the pseudocolor plot of the TA spectrum and the respective cross sections at different time delays (Δ*t*) are presented in Figure 2b. It can be seen that in the early timescales (i.e., Δ*t* < 0.5 ps), the spectral data features an asymmetric derivative shape with photoinduced redshifted absorption, which reflects the bandgap renormalization (BGR) process induced by many‐body Coulomb interaction among photoexcited carriers.^[^ ^16^, ^35^, ^36^ ^]^ As time progresses, the derivative lineshape is replaced by a strong bleaching feature and then, a weak/broad photoinduced absorption (PIA) band gradually develops toward the low energy side. According to the nanosecond carrier lifetime in IMPB,^[^ ^26^ ^]^ the carrier population should remain constant on the subpicosecond time scale and as a result, we can attribute the disappearance of the BGR effect to the formation of polarons, which will screen the initial many‐body Coulomb interactions.^[^ ^16^, ^36^ ^]^ Meanwhile, the emergence of a PIA band at the low energy side is also an important optical fingerprint for the formation of polaronic states considering the activation of forbidden middle‐gap transitions from polaronic ground states to the conduction band.^[^ ^36^, ^37^, ^38^ ^]^ It is worth mentioning that based on the TA results of Mohammed et al.,^[^ ^38^ ^]^ such a polaron‐induced PIA feature could only be observed in LHPs with soft octahedral (i.e., the individual octahedra are easily perturbed by the photoexcitation), consistent with large degrees of freedom in IMPB.^[^ ^26^ ^]^

Besides the above‐mentioned features, we have noticed that the negative bleaching band of IMPB in Figure 2a,b exhibits a bimodal behavior (i.e., the bleaching is splitting into two bands, denoted as PIB‐I and PIB‐II). We have plotted the maximum amplitude of PIB‐II at ≈5 ps as a function of photoexcitation carrier density in Figure 2c and the amplitude increases linearly without any saturation signature even at *N*_0_ above 10^19^ cm^−3^, which suggests the intrinsic nature^[^ ^39^, ^40^ ^]^ of such a band splitting and is consistent with PL results (i.e., low density of defects and vacancies). Recently, the dynamic Rashba effect has been observed in halide perovskites (i.e., strong spin‐orbit couplings with a built‐in electric field induced by an ultrashort pumping pulse could transiently break structural inversion symmetry),^[^ ^41^, ^42^, ^43^ ^]^ which transforms the conduction band into upper/lower subbands and splits the TA peak.^[^ ^44^ ^]^ However, it is well‐established that the Rashba split energy should be linearly dependent on the strength of the transient electric field (i.e., photoexcited carrier density),^[^ ^43^, ^44^, ^45^ ^]^ which is highly against the declined trend observed in IMPB (see the blue diamonds in Figure 2c and the spectra cuts at Figure S3, Supporting Information). Considering the long‐range ferroelectricity, we argue that the strong internal electric field (i.e., the electric field E = 5.7 × 10^7^ V cm^−1^ is calculated from *P*_s_ = ≈5.0 µC cm^−2^) could polarize the polarons in IMPB and cause the Stark splitting of the corresponding transitions, as previously reported in other ferroelectric materials.^[^ ^46^, ^47^ ^]^ This argument can be further justified by the following experimental facts: (i) The Stark split energy is only related to the internal electric field, which will be partially screened by the photoexcited carriers and shows a decreased behavior with higher *N*_0_; (ii) Consistent with Stark splitting in other materials,^[^ ^46^, ^48^ ^]^ PIB‐I and PIB‐II transitions are both transition‐allowed and emissive, as shown in the streak camera recorded time‐resolved PL (see Figure S4, Supporting Information).

**Figure 3:**
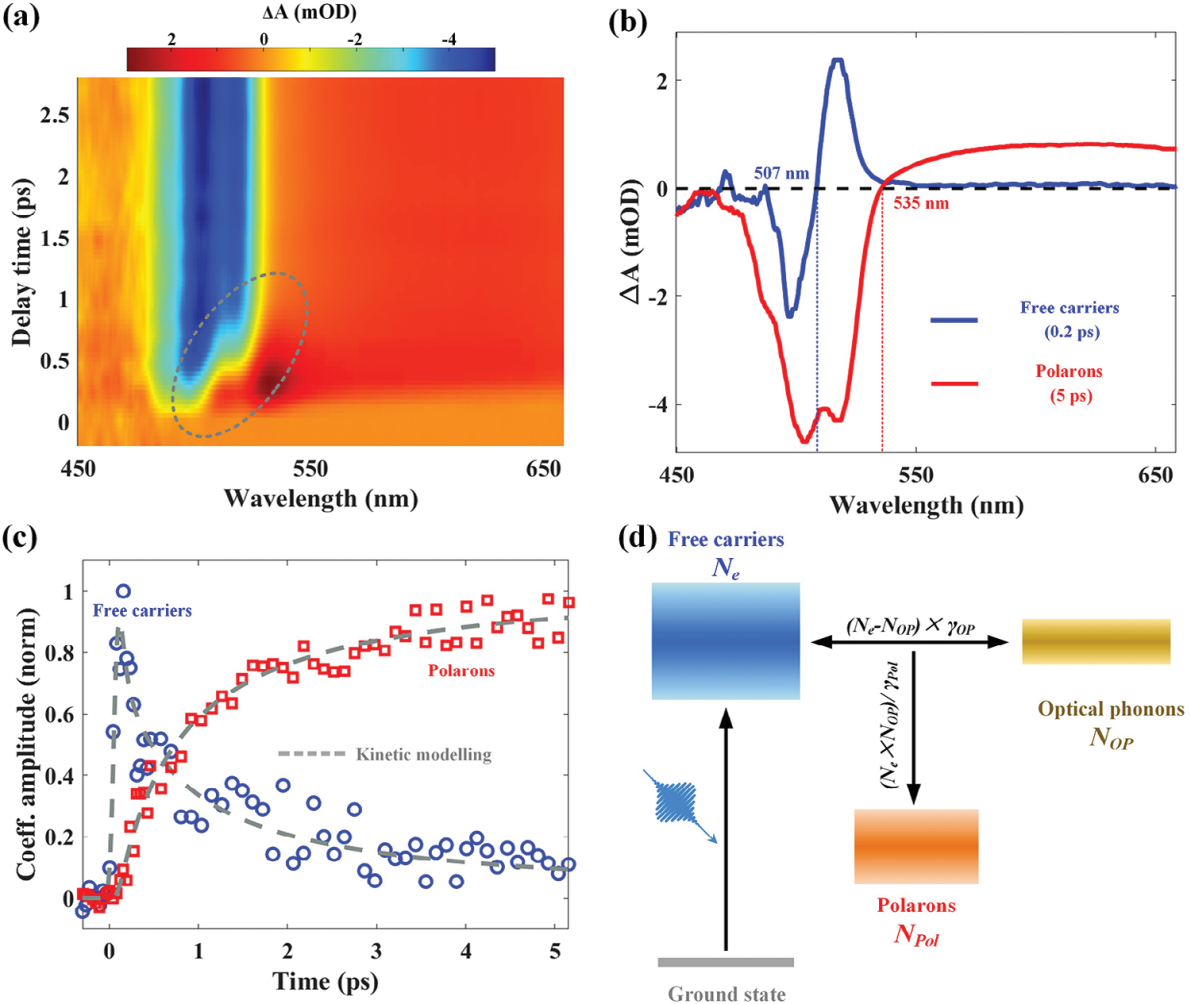
The formation dynamics of polarons in IMPB with the excitation carrier density of ≈1.9 × 10^18^ cm^−3^ and the excitation wavelength of 360 nm. a) The 2D TA map in the short time range, which emphasizes the red‐shifting of the transition edge right after photoexcitations. b) Representative spectra at early (denoted free carriers, ≈0.2 ps) and later (denoted polarons, ≈5 ps) delay times. c) Time dynamics of the initial state (free carriers) and the final state (polarons) from the multivariable regression. The dashed lines are fitting results using a two‐temperature model. d) Schematic illustration of the two‐temperature model for polaron formation.

Since the Coulombic interactions are screened by polarons, the transition edge (i.e., the zero‐crossing) provides a convenient reference to track the polaron formation process in IMPB,^[^ ^40^, ^49^, ^50^ ^]^ which is circled for clarity in **Figure** **3**a. Following Leone et al.,^[^ ^50^ ^]^ we have compared the characteristic TA spectra at the early time (≈0.2 ps) right after photoexcitation and at a later time (≈5 ps), after which the spectral shape remains nearly unchanged. As shown in Figure 3b, the zero‐crossing energy redshifts from ≈507 to ≈535 nm within the first 5 ps, indicating the transition of photogenerated free carriers to polarons (i.e., Lindenberg et al. have demonstrated that zero‐crossing energy shift in LHPs can be linked to a time‐dependent change in the effective mass of carriers^[^ ^49^ ^]^). To quantitatively determine the formation dynamics while avoiding the spectral overlap, we have extracted the kinetics from the TA spectra by using a multivariable regression taken at ≈0.2 ps (the initial state: free carriers) and ≈5 ps (the final state: polarons). Time‐resolved amplitudes decomposed from the multivariate regression are shown in Figure 3c as blue circles and red squares for free carriers and polarons, respectively. It can be seen that the amplitude of free carriers rises within the excitation pulse width (≈100 fs), then decays closely following the amplitude rising of polarons with a similar rate till several picoseconds, confirming again the transfer of free carriers to polarons in IMPB.

To account for the role of optical phonons in the formation of polarons in IMPB, we have modeled the amplitude versus time in Figure 3c using the two‐temperature kinetic model (TTM)^[^ ^51^, ^52^ ^]^ instead of traditional exponential fittings. As schematically shown in Figure 3d, this kinetic model allows bimolecular recombination between the free carrier and the optical phonon to create a polaron, which thus yields two rate constants (i.e., electron‐phonon scattering and polaron formation) and two amplitudes (i.e., the average populations of free carriers and polarons), respectively. Detailed discussions of the TTM model and corresponding rate equations are provided in Figure S5, Supporting Information. Please note that in the TTM model, we do not consider the scattering with acoustic phonons since the nonpolar nature of acoustic phonons contributes negligible in this short time scale (<10 ps)^[^ ^53^, ^54^ ^]^ and the impact of multiple optical phonon scatterings arising from excess carrier energy has also been ruled out based on the overlapping of dynamic populations between free carriers and optical phonons in Figure S6, Supporting Information. The TTM fitting in Figure 3c (the dashed lines) gives a carrier‐phonon scattering time of 320 ± 38 fs and a polaron bimolecular formation time of 1140 ± 15 fs, which are largely prolonged compared to the value reported in non‐ferroelectric LHPs (i.e., inter‐/intra‐valley electron‐phonon scatterings occur in 30–60 fs^[^ ^55^ ^]^ and polaron formation times is less than 1 ps^[^ ^56^ ^]^). This distinction is expected in ferroelectric materials since the presence of long‐range polarization can screen the Coulomb effect, reducing electronic interaction with lattice vibrations and thus slowing down carrier‐phonon scatterings.^[^ ^24^, ^57^ ^]^

**Figure 4:**
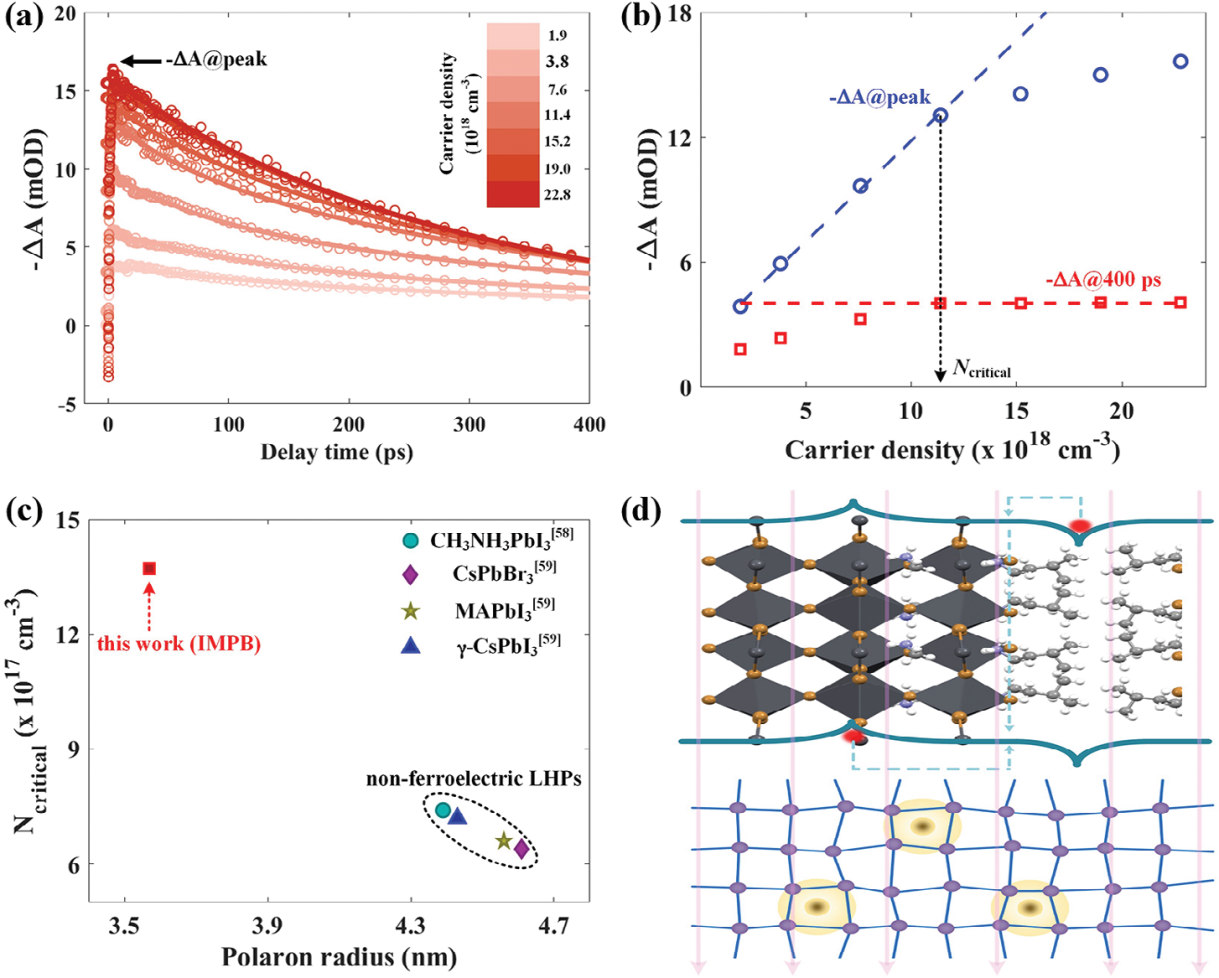
Carrier density‐dependent polaron dynamics in IMPB with the excitation wavelength of 360 nm. a) Time‐resolved –ΔA signals with different carrier densities. b) The amplitude of –ΔA signals at the peak and at the delay time of 400 ps as a function of the carrier density. The dashed line is a linear fitting to determine the Mott density. c) Comparison of the Mott polaron density and the corresponding polaron radius between different LHPs. d) A schematic illustration of the polaron size evolution with macroscopic polarizations of ferroelectricity.

The impact of ferroelectricity on the polaron can also be investigated based on the Mott polaron density (*N*_critical_), which is proposed by Frost et al.^[^ ^58^ ^]^ and defined as the critical carrier density where polaron wavefunctions overlap. **Figure** **4**a has shown the fluence‐dependent polaron decaying dynamics in IMPB (i.e., the polaron dynamics in the time window of several hundred picoseconds) with varying photoinjected carrier density *N* from ≈1.9 × 10^18^ to ≈2.3 × 10^19^ cm^−3^ (see the evolution process of *N* in Experimental Section and the *N*‐dependent 2D color plot of TA spectra in Figure S7, Supporting Information). It can be seen that with low carrier densities, the peak value of polaron dynamics increases linearly and shows little decay within our time window of ≈400 ps, in line with our above argument that long‐range polarization screens the Coulomb effect and prolongs the polaron dynamics. While with high carrier densities and the enhanced wavefunction overlapping among polarons, the dynamics exhibit distinct features with a sub‐linear peak‐increasing trend and an accelerated decaying process,^[^ ^58^, ^59^ ^]^ which brings the –ΔA signal quickly to the same level at ≈400 ps, independent of the initial photoinjected carrier density (also see the individual polaron dynamics with exponential fittings in Figure S8, Supporting Information). This *N*‐dependent polaron relaxation has been further presented in Figure 4b by plotting the –ΔA signal at two representative time cuts (i.e., at the peak or ≈400 ps), in which the linear relation between the peak value (the blue dashed line) and the plateau points at ≈400 ps (the red dashed line) defines *N*_critical_ in IMPB as 1.14 × 10^19^ cm^−3^.

With the Mott polaron density, we can now estimate the polaron radius (*r*_pol_) in IMPB by defining the polaron overlapping when each polaron occupies a cube with the side twice its radius and thus, *N*_critical_ can be related to *r*_pol_ as:^[^ ^58^, ^60^ ^]^ *N*_critical_ = (2 × (2 × *r*_pol_)^3^)^−1^. Here, a factor of 2 is accounting for the capture of both holes and electrons. In Figure 4c, we have correlated the experimentally extracted *N*_critical_ in IMPB and reported *N*_critical_ in non‐ferroelectric LHPs^[^ ^58^, ^59^ ^]^ to the polaron radius. The estimated polaron radius in non‐ferroelectric LHPs (e.g., CH_3_NH_3_PbI_3_, CsPbBr_3_, MAPbI_3_, γ‐CsPbI_3_) is ≈5 nm, which is consistent with theoretical values based on the Feynman polaron model,^[^ ^61^, ^62^ ^]^ verifying again that neighboring polarons overlap at *N*_critical_. While for ferroelectric IMPB, the large *N*_critical_ renders a relatively small polaron radius of ≈3.6 nm, implying the Frohlich interactions may be sufficiently screened by the internal polarizations (polar lattices) in ferroelectric semiconductors. As schematically shown in Figure 4d, the ferroelectric polarization in IMPB produces much wider and deeper potential wells, which localizes carriers into polar nanodomains and introduces efficient charge carrier screening, leading to long charge carrier lifetimes and reduced distributions of polaron wavefunctions. In principle, the photophysics of ferroelectric semiconductors could contribute to improved optoelectronic properties considering that the stable Mott state at higher carrier densities impedes the efficient phonon diffusion away from the hot carriers, which, in turn, may maintain hot carriers for a long time for device harvesting.

Altogether, our works experimentally investigate new photophysics in carrier dynamics of ferroelectric LHPs. Specifically, we, for the first time, (i) demonstrated the impact of macroscopic ferroelectricity on polarons in LHPs by revealing three anomalous dynamic features, which were not observed or aware in normal non‐ferroelectric LHPs before; (ii) provided clear explanations of the mechanism of anomalous polarons in ferroelectric LHPs by combining the detailed transient spectroscopy measurements and kinetic modeling; (iii) Observed a giant polaron Mott density in ferroelectric LHPs (i.e., over an order of magnitude higher than non‐ferroelectric counterparts), which may render potential applications in high‐excitation optoelectronic applications. Therefore, this work has a certain pioneering and enlightening nature regarding the photophysics of ferroelectric LHPs, which also have strong implications for their applications in LEDs, lasing, and concentrator photovoltaics. Moreover, these findings will also play an important role in providing critical knowledge to broaden the understanding of ferroelectric polaron in LHPs beyond the current regime of transient polarizations.

## 3 Conclusion

In conclusion, our comprehensive time‐resolved spectroscopy investigation has unveiled intriguing polaron dynamics in IMPB exhibiting long‐range ferroelectricity. Specifically, we have elucidated anomalous features in the polaron dynamics of IMPB, including a slower polaron formation process (i.e., the time constant of ≈1.1 ps in IMPB versus ≈0.3 ps in non‐ferroelectric LHPs), an unexpected splitting of the PIB exceeding 60 meV, and a Mott polaron density more than one order of magnitude larger (i.e., *N*_Mott_ of ≈7.6 × 10^18^ cm^−3^ in IMPB versus *N*_Mott_ of 3–6 × 10^17^ cm^−3^ in non‐ferroelectric LHPs). These observations are attributed to the persistent and unidirectional polarizations inherent in ferroelectric perovskites, leading to a suppressed carrier‐phonon scattering and an internal Stark effect. Our findings provide insight into the intricate and dynamic behavior of polarons in quantum‐confined ferroelectric materials, opening new prospects for the design of desirable optoelectronics.

## 4 Experimental Section

### Synthesis of (IA)_2_(MA)_2_Pb_3_Br_10_

The lead acetate trihydrate (20 mmol, 7.59 g), methylamine (10 mmol, 0.77 g), and isoamylamine aqueous solutions (20 mmol, 2.02 g) were weighed successively and slowly added to the solution. Under the heating condition of 100 °C, the clarified solution was obtained after continuous stirring for ≈30 min, with obtaining high‐quality particle seed crystals after cooling to room temperature. Subsequently, the seed crystals were immersed in the solution and large‐scale crystals were grown through the top seed growth method. Finally, the yellow flake crystals were obtained in the saturated solution through a temperature‐cooling process.

### DFT Calculations of Electronic Structure

Based on the CASTEP^[^ ^63^ ^]^ and the ferroelectric single‐crystal structural data at room temperature,^[^ ^26^, ^27^ ^]^ we used Perdew‐Burke‐Ernzerhof generalized gradient approximation to evaluate the exchange‐correlation effects and utilized OTFG Ultrasoft pseudo‐potential to describe the core‐electrons interactions.^[^ ^64^ ^]^ The integration of the Brillouin zone was performed using a Monkhorst‐Pack κ‐point sampling of 6 × 6 × 3 and the numbers of plane waves included in the basis sets were determined by an energy cutoff of 765 eV. The crystal structure is optimized through the Broyden‐Fletcher‐Goldfarb‐Shanno algorithm with the following parameters: the convergence criterion for maximum atomic displacement was set as 5 × 10^−4^ Å, the convergence criterion for the interatomic force was set as 0.01 eV Å^−1^, the convergence criterion for stress within the crystal was set as 0.02 GPa, and the convergence criterion for energy of a single atom was set as 5 × 10^−6^ eV. As the generalized gradient approximation underestimates the energy band gap,^[^ ^65^ ^]^ the hybrid functional HSE06^[^ ^66^ ^]^ was employed to calculate the accurate electronic properties of the IMPB.

### Transient Absorption Spectroscopy

Femtosecond‐resolved TA measurements are performed using a commercial spectrometer. The pump pulse is generated from an optical parametric amplifier that is pumped by a 1 kHz regenerative amplifier (80 fs, 1 kHz, 800 nm), which is seeded by mode‐locked Ti‐sapphire oscillators (80 MHz). The white light continuum probe beam (in the range from 400 to 1500 nm) is generated by focusing a small portion (≈10 µJ) of the regenerative amplifier's fundamental 800 nm laser pulses into either a 2 mm sapphire crystal (for visible range) or a 1 cm sapphire crystal (for NIR range). The probe beam is collected using a CMOS sensor for the ultraviolet‐visible region.

### Estimation of the Carrier Density

The initial carrier density (*N*_0_) is calculated based on the Beer‐Lambert law: *N*_0_ = *F* × (1–10^−^ *^A^*)/*E*_ph_/*d*, where *F* is the excitation fluence with the pump spot radius of ≈55 µm which is determined by using the knife‐edge method, *A* of is the absorbance at the excitation wavelength of 360 nm, *E*_ph_ is the excitation photon energy (360 nm, 3.44 eV), and *d* is the laser penetration depth in IMPB at the excitation wavelength of 360 nm.

## Conflict of Interest

The authors declare no conflict of interest.

## Supporting information



Supporting Information

## Data Availability

The data that support the findings of this study are available from the corresponding author upon reasonable request.
