# Supplementary material for: Anomalous Polarons in Two‐Dimensional Organometallic Perovskite Ferroelectric
Source: Adv Sci (Weinh). 2024 Sep 23;11(43):2406885. doi: 10.1002/advs.202406885 (PMC11578327; doi:10.1002/advs.202406885)
Supplement: Supplementary file 1 — Supporting Information [file ADVS-11-2406885-s001.pdf]

ADVANCED SCIENCE
Open Access

Supporting Information

for *Adv. Sci.*, DOI 10.1002/advs.202406885

Anomalous Polarons in Two-Dimensional Organometallic Perovskite Ferroelectric

Junhong Yu, Yadong Han, Yunfan Yang, Hang Zhang, Yi Liu, Jinlong Xu, Zhihua Sun and Jianbo Hu**

SUPPLEMENTARY INFORMATION

Anomalous Polarons in Two-Dimensional Organometallic Perovskite Ferroelectric

Junhong Yu,[1] Yadong Han,[1,2] Yunfan Yang,[1,2] Hang Zhang,[1,2] Yi Liu,[3] Jinlong Xu,[4] Zhihua Sun,[3,#] Jianbo Hu[1,2,#]

[1]Laboratory for Shock Wave and Detonation Physics, Institute of Fluid Physics, China Academy of Engineering Physics, Mianyang 621900, China

[2]State Key Laboratory for Environment-Friendly Energy Materials, Southwest University of Science and Technology, Mianyang 621010, China

[3]State Key Laboratory of Structural Chemistry, Fujian Institute of Research on the Structure of Matter, Chinese Academy of Sciences, Fuzhou, Fujian 350002, China

[4]National Laboratory of Solid State Microstructures, School of Electronic Science and Engineering, School of Physics, Nanjing University, Nanjing 210093, China

[#]To whom correspondence should be addressed. Email: sunzhihua@fjirsm.ac.cn (ZS); jianbo.hu@caep.cn (JH)

Supplementary Figure 1

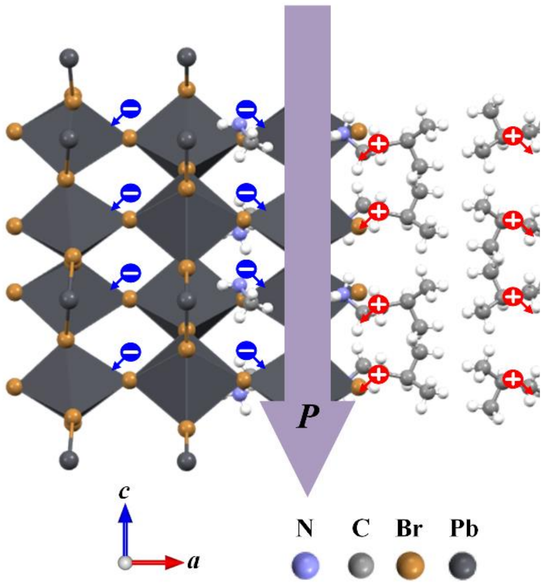

Figure S1. A cartoon to illustrate the ferroelectric polarization in IMPB. From the packing views projected along the *b*-axis at room temperature, the MA^+ cation alignment inside the inorganic sheets created a dipolar component along the *c*-axis, and the bulky IA^+ cations exhibited the same orientation, which together shift positively charged centers in the *c*-direction. While for the $[Pb_3Br_{10}]_\infty$ sheets, the corner-sharing $PbBr_6$ octahedra presented a distorted geometry, which accounted for the displacement of negatively charged centers along the *c*-axis. The separation of positive and negative charges thus favors the appearance of ferroelectric polarization in the c-axis direction.

Supplementary Figure 2

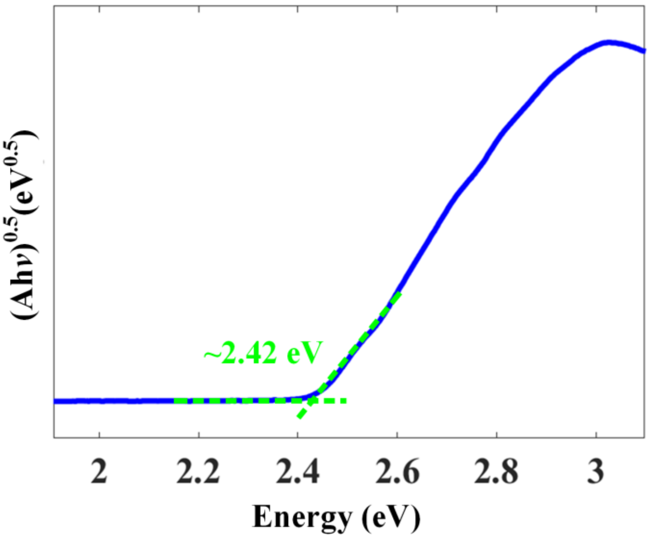

Figure S2. The Tauc plot for IMPB. The normalized absorbance (A) measured in Fig. 1c is used for the $(Ah\nu)^{0.5}$ term on the vertical axis and the optical bandgap is determined using the Tauc equation[1], which yield the bandgap of ~2.42 eV, consistent with our DFT calculations.

Supplementary Figure 3

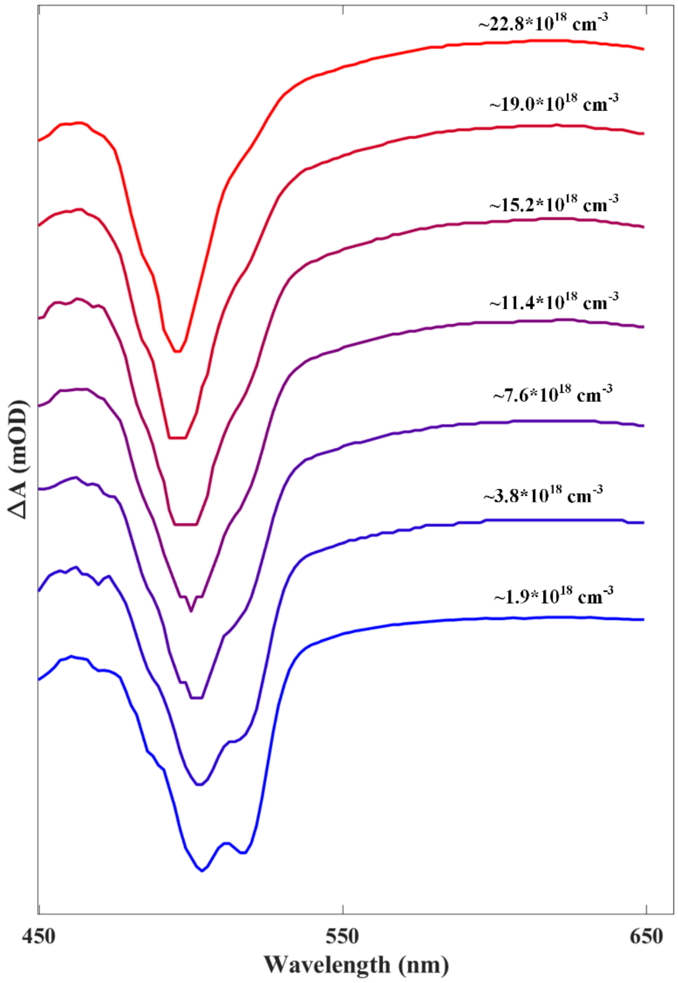

Figure S3. The spectra cut of transient absorption measurements at ~5 ps with different excitation carrier densities. It can be seen that the separation energy between the PIB-1 and PIB-2 features are decreasing with the increased carrier density.

Supplementary Figure 4

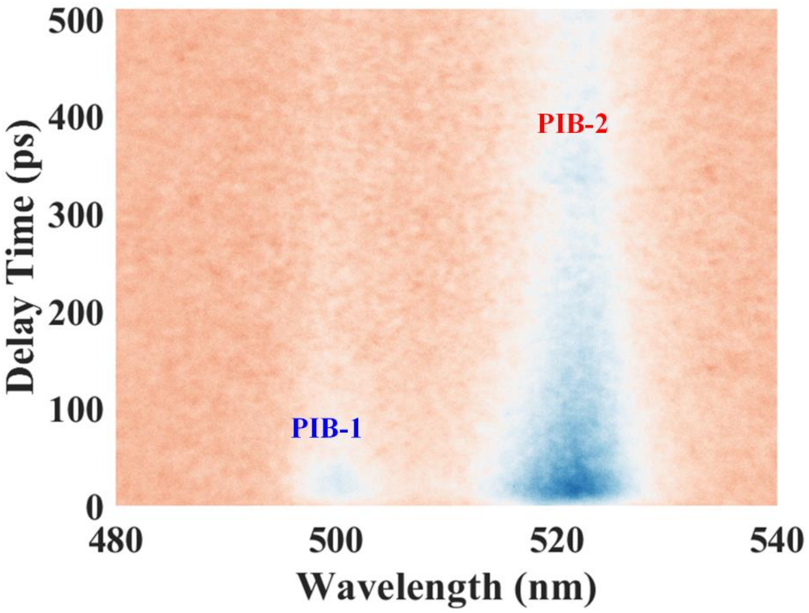

Figure S4. Time-resolved PL of IMPB at room temperature recorded by the streak camera. The PIB-I and PIB-II are both emissive and transition-allowed.

Supplementary Figure 5

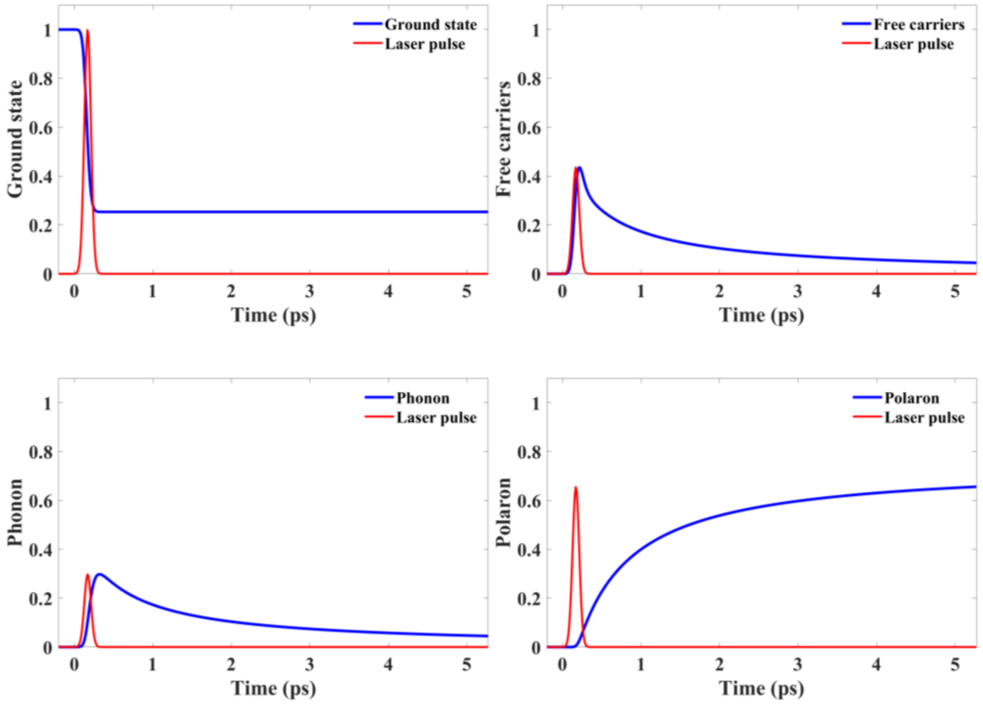

Figure S5. Simulated population dynamics based on the TTM rate equation model. The red curve is the excitation laser pulse with a pulse duration of 100 fs. The carrier density is ~1.9×10^{18} cm^{-3}.

Since the polaron formation dynamics reflect localization of the photoexcited free carriers, a two-temperature rate equation model[2-4] is adapted to describe the carrier-phonon interaction and the polaron formation process in IMPB:

$$\frac{dN_e}{dt} = G(t) - \frac{N_e - N_{phonon}}{\tau_{phonon}} - \frac{N_{phonon} N_e}{\tau_{polaron}}$$

$$\frac{dN_{phonon}}{dt} = \frac{N_e - N_{phonon}}{\tau_{phonon}} - \frac{N_{phonon} N_e}{\tau_{polaron}}$$

$$\frac{dN_{polaron}}{dt} = \frac{N_{phonon} N_e}{\tau_{polaron}}$$

Where *N* is the population, *G(t)* represents the excitation source, and τ is the lifetime.

In this kinetic model, photoexcitation first creates a hot free carrier population. Then, a non-thermal optical phonon population is created due to the carrier-phonon scattering, and the energy transfer time between the lattice/electronic subsystem is characterized by τ_{phonon}. Meanwhile, in addition to carrier-phonon scattering, an optical phonon and an electron can recombine to localize in a polaron. Therefore, using this model to fit the experimental data, we can extract the polaron formation dynamics can be extracted.

Supplementary Figure 6

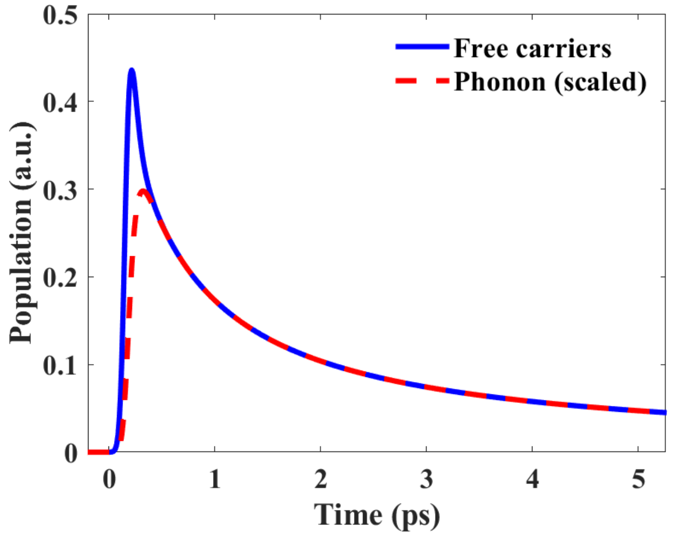

Figure S6. Comparing the calculated polaron dynamics (the blue solid line) and phonon dynamics (the red dashed line). Here the phonon effective population is scaled the same as the free carrier population at long delay times.

Please note that in the kinetic equation model, we do not consider the free carrier and optical phonon scattering with acoustic phonons because of the low temperatures (<350 K) and short time scales (<10 ps) modeled[5,6]: in our modeling condition, only the polar optical phonons take part in polaron formation and acoustic phonons would not be expected to directly change the polaron formation rate. Also in this model, polaron formation is treated statistically, ignoring the spatial nature of the free carrier and phonon (i.e., dynamic diffusion process) that creates the polaron after photoexcitation.

Although the optical phonon population is not directly measured in our experiment, for visualization it is shown as a dashed line in **Fig. S6** scaling the same amplitude as the electron population It can be seen that despite the deviation during the first 100 fs excitation pulse duration, the free carrier dynamics is very similar to the optical phonon dynamics, implying that in IMPB, the polaron formation is mainly coming from one-phonon scattering process (i.e, one free carrier scattering to create one phonon, which then recombines to create one polaron)

Supplementary Figure 7

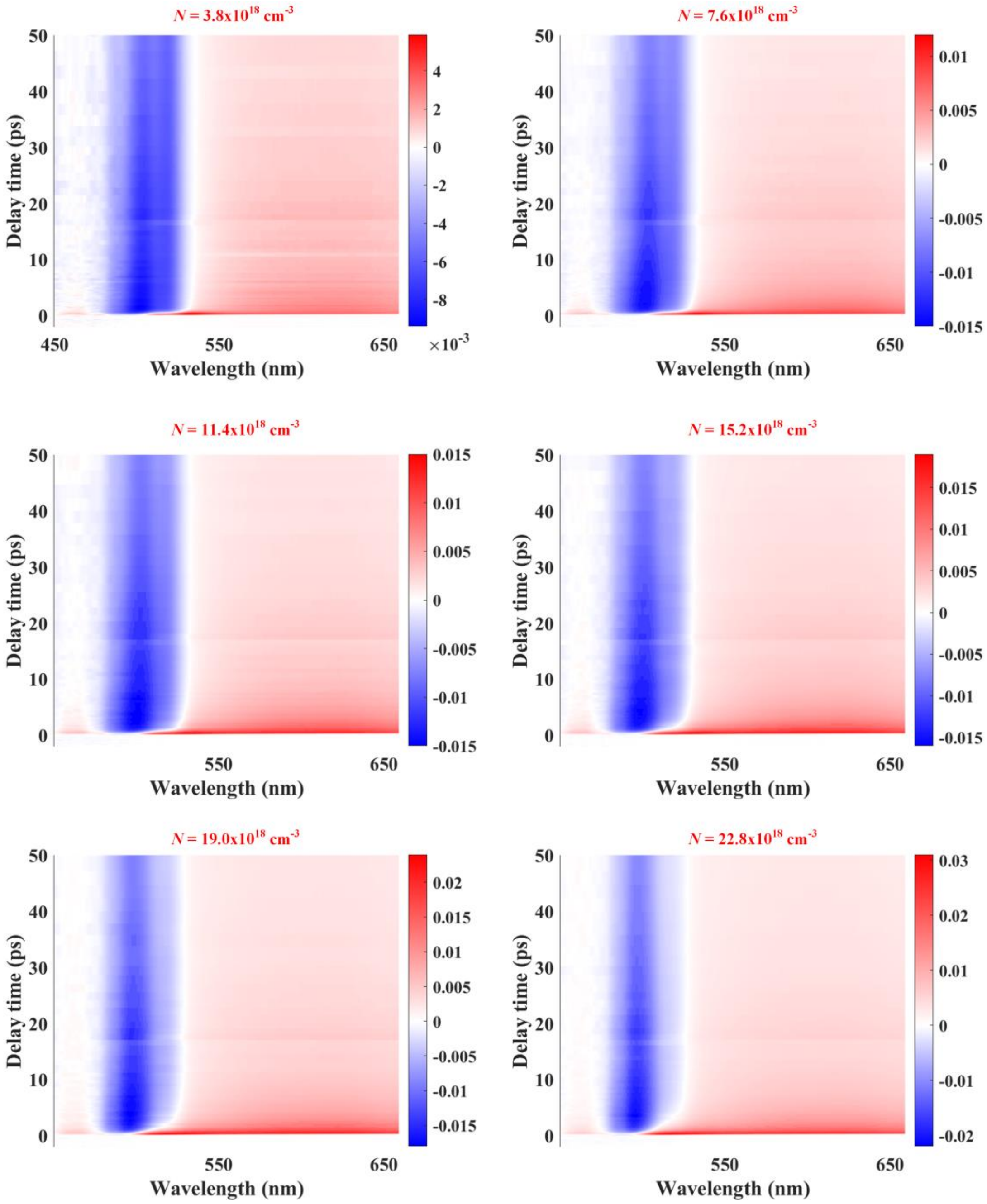

Figure S7. 2D color plots of TA spectra with different carrier densities.

Supplementary Figure 8

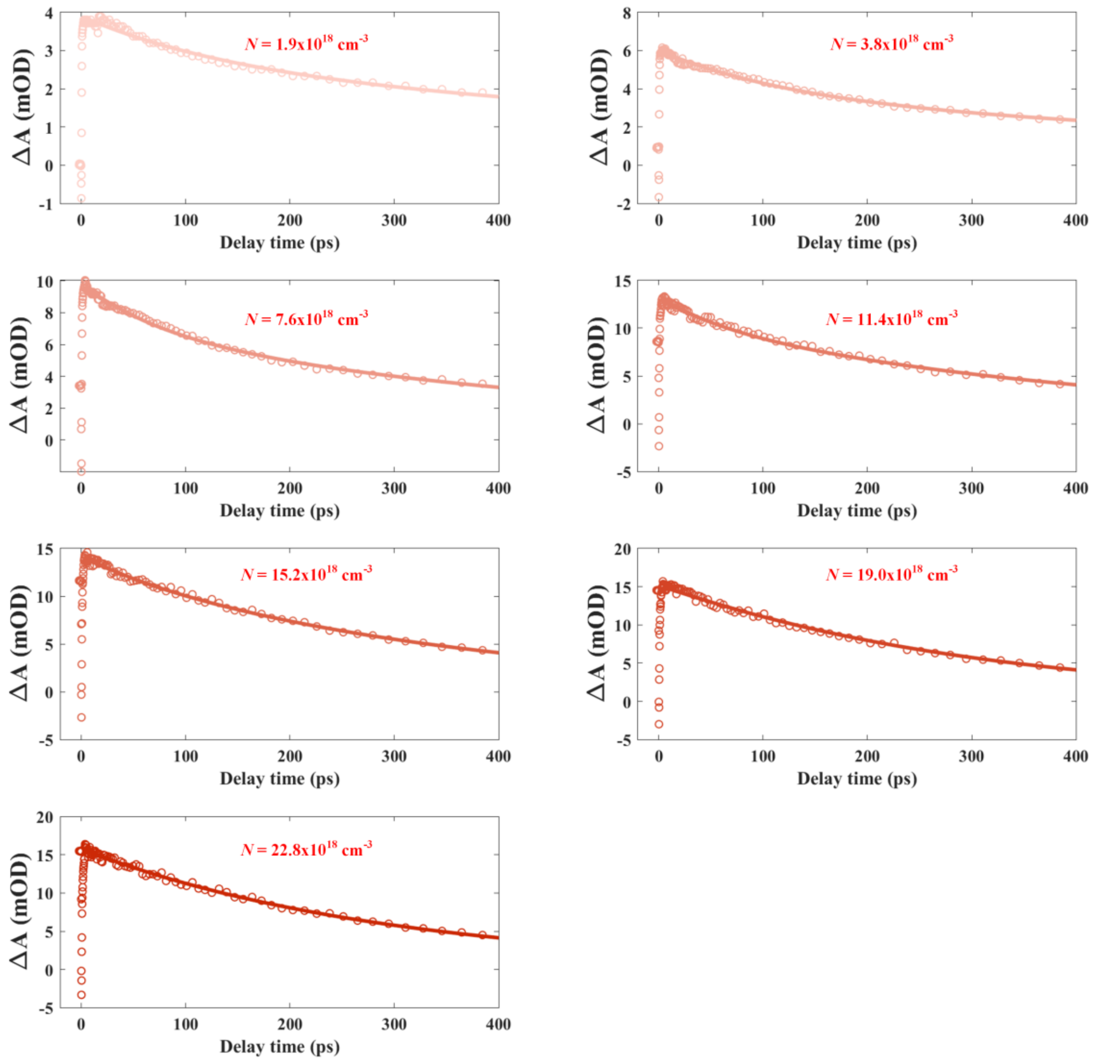

Figure S8. Polaron dynamics of the IMPB crystal with different carrier densities (the solid black line is fit for polaronic kinetics using a bi-exponential function).

A photoinduced positive broadband appears above 535 nm in the spectrum of the IMPB crystal owing to polaron absorption. As shown in **Fig. S8**, the corresponding kinetics of the polaron decaying dynamics is fitted by a bi-exponential function convoluted with a Gaussian instrument response (τ_0, 100 fs)[4]:

$$\Delta A(t) = A_1 \bullet erf\left(\frac{t-t_0}{\tau_0} - \frac{\tau_0}{2\tau_1}\right)\exp\left(-\frac{t-t_0}{\tau_1}\right) + A_2 \bullet erf\left(\frac{t-t_0}{\tau_0} - \frac{\tau_0}{2\tau_2}\right)\exp\left(-\frac{t-t_0}{\tau_2}\right)$$

Reference

1. Prasanna, R. et al. Band gap tuning via lattice contraction and octahedral tilting in perovskite materials for photovoltaics. *J. Am. Chem. Soc.* **139**, 11117–11124 (2017).

2. Carneiro, L. M. et al. Excitation-wavelength-dependent small polaron trapping of photoexcited carriers in *alpha*-Fe_2O_3. *Nat. Mater.* **16**, 819-825 (2017).

3. Yu, J. et al. Electrically control amplified spontaneous emission in colloidal quantum dots. *Sci. Adv*. **5**, eaav3140 (2019).

4. Yu, J.; Sharma, M.; Sharma, A.; Delikanli, S.; Demir, H. V.; Dang, C. All-optical control of exciton flow in a colloidal quantum well complex. *Light: Sci. Appl.* **2020**, 9, 27.

5. Yu, J. et al. Intrinsic Self-Trapped Excitons in Graphitic Carbon Nitride. *Nano Lett.* **24**, 4439-4446 (2024).

6. Yu, J. et al. Observation of Phonon Cascades in Cu-Doped Colloidal Quantum Wells. *Nano Lett.* **22,** 10224–10231, (2022).
